# Blind Estimation Versus Direct Measurement of the Arterial Input Function in Dynamic Contrast‐Enhanced MRI of the Breast

**DOI:** 10.1002/mrm.70454

**Published:** 2026-06-12

**Authors:** Jake L. Cray, Jiří Vitouš, Radovan Jiřík, Sofya Titarenko, David L. Buckley

**Affiliations:** ^1^ University of Leeds Leeds UK; ^2^ Institute of Scientific Instruments, Czech Academy of Sciences Brno Czech Republic; ^3^ Faculty of Electrical Engineering and Communication Brno University of Technology Brno Czech Republic

**Keywords:** arterial input function, breast cancer, dynamic contrast‐enhanced MRI, perfusion

## Abstract

**Purpose:**

Accurate arterial input functions (AIFs) are essential for quantitative dynamic contrast‐enhanced (DCE) MRI, yet direct measurement is challenging and population‐averaged AIFs neglect patient‐specific variability. Blind deconvolution provides an alternative by estimating patient‐specific AIFs directly from tissue data, up to a scale factor. This study compared blindly estimated AIFs with carefully measured aortic AIFs in breast DCE‐MRI.

**Theory and Methods:**

Data from 25 patients with breast cancer were analyzed, each with a carefully measured AIF. Blind AIF estimates were obtained using model‐constrained deconvolution in the Perflab toolkit with both Tofts–Kety (TK) and two‐compartment exchange (2CXM) tissue models. To help isolate AIF shape blind AIFs were scaled using cardiac output. These blind AIFs were compared with measured AIFs using scale‐invariant and scale‐dependent metrics which assess the similarity of the AIF shape.

**Results:**

Blind estimates were obtained for all 25 patients with both tissue models. Compared with measured AIFs, blind estimates derived using the 2CXM showed stronger agreement across all metrics than those derived using the TK model. However, the weak correlation in scale‐dependent metrics suggests limitations of cardiac output scaling.

**Conclusion:**

Blind AIF estimation using the 2CXM provides more reliable recovery of AIF shape and dispersion than the TK model in breast DCE‐MRI. While blind deconvolution shows promise for estimating local patient‐specific AIFs other scaling strategies may need to be employed in practice.

## 1 Introduction

$T_{1}$‐weighted dynamic contrast‐enhanced (DCE)‐MRI is widely used to assess tissue and tumor hemodynamics, including perfusion, and capillary permeability. Quantitative analysis of DCE‐MRI requires an arterial input function (AIF), which describes the contrast agent (CA) concentration in a feeding artery over time. Ideally, the AIF would be measured in an artery close to the tissue of interest. However, in practice this is often not feasible. Additionally, accurate AIF measurement is subject to several limitations, including arteries falling outside the field of view (FOV), $T_{2}$* effects (which reduce the peak height of the AIF), partial volume effects, inflow artifacts, low temporal resolution, and limited signal‐to‐noise ratio (SNR) [1, 2]. As a compromise, population‐averaged AIFs are often used to approximate a typical input. Although, these offer repeatable estimates of perfusion parameters, they inherently bias results by neglecting patient‐specific variation in AIFs [3]. More recently, blind deconvolution has gained traction as a technique for estimating patient‐specific AIFs directly from tissue data [4, 5, 6, 7, 8, 9, 10, 11, 12]. While these approaches show promising results in simulations, validation on clinical data remains challenging because of the measurement issues outlined above.

An advantage of blind deconvolution is that the estimated AIF is local to the tissue region of interest (ROI), as if measured from an artery directly supplying that tissue [13]. In contrast, use of a global AIF, measured in a large upstream artery such as the descending aorta, can introduce systematic errors into quantitative analysis. Compared with global AIFs, local AIFs are expected to be delayed and dispersed [1]. A further limitation of blind deconvolution is that AIFs are estimated only up to a scale factor, so a secondary scaling step is required.

In this study, we assess the performance of an existing implementation of blind deconvolution [12, 14] by comparing blind AIFs with measured AIFs obtained from a breast DCE‐MRI dataset that was specifically designed to measure AIFs in the descending aorta while minimizing common error sources [2].

Our comparisons between AIFs use both scale‐invariant and scale‐dependent metrics. For scale‐dependent metrics, cardiac output (CO) scaling is applied, with the blind AIF scaled according to the inverse relationship between the area under the first‐pass peak and CO [15]. The use of CO has been previously demonstrated to improve AIF measurement quality in [15] and used in the context of blind deconvolution in [6]. This is not feasible in studies without a measured AIF (or a direct measurement of CO). Here it is done to isolate the shape of the AIFs for comparison.

The primary aim of this study was to evaluate blind estimation of AIFs in breast DCE‐MRI by comparing the shapes of blindly estimated AIFs obtained using the one‐compartment Tofts–Kety (TK) and two‐compartment exchange (2CXM) models [16] with measured aortic AIFs.

## 2 Methods

### 2.1 Modeling

*Horsfield AIF model*: We modeled the AIF using a parametric functional form to constrain the blind deconvolution, imposing prior knowledge about the expected curve shape. This also allows direct comparison between parameters from blindly estimated AIFs and those obtained by fitting the same model to the measured AIFs. We used a functional form described in [17]:

(1) $$C_{p}(t)=\overset{3}{\underset{i=0}{\sum }}A_{i}e^{-m_{i}t}\overset{n}{\underset{j=0}{\sum }}\gamma ((j+1)\alpha +j,\;\beta ,\;t-j\tau ),$$

which is evaluated piecewise over nτ<t<(n+1)τ, where n=⌊t/τ⌋. Here, n is not a free parameter, but an integer index that counts the number of completed recirculations at time t. All quantities $A_{i}$, $m_{i}$, α, β, τ are free parameters in the model. Specifically $A_{i}$ and $m_{i}$ are amplitude and decay constants, α and β are positive shape and scale parameters for the gamma variate function

(2) $$\gamma (\alpha ,\beta ,t)=\frac{t^{\alpha }e^{-t/\beta }}{\beta^{\alpha +1}\;\Gamma (\alpha +1)},\;t\geq 0,$$

τ is the minimum recirculation time (for the CA to travel around the vascular system), and n specifies the number of recirculations measured. In practice, we evaluate the gamma variate function in log‐space:

(3) $$\begin{array}{cc} \log\gamma (\alpha ,\beta ,t) & =\alpha \log t-\frac{t}{\beta }\\ & \;-(\alpha +1)\log\beta -\log\Gamma (\alpha +1),\;t>0. \end{array}$$

This avoids numerical overflow for large t, due to the terms $t^{\alpha }$ and $e^{-t/\beta }$. The normalization constant Γ(α+1) is computed via logΓ(α+1), which is numerically more stable. This approach also reduces computational cost.

*Tissue models*: Tissue residue functions (TRFs) are also required in order to predict tissue concentration curves and compute residuals for the minimization problem. We use a one‐compartment model (TK) and two‐compartment exchange model (2CXM), which are described in [18]:

(4) $$\begin{array}{cc} R_{\text{TK}}(t) & =F_{p}e^{-(F_{p}/\;(v_{p}+v_{e}))t},\\ R_{\text{2CXM}}(t) & =F_{+}e^{-k_{1}t}+F_{-}e^{-k_{2}t},\\ & =F_{p}\left[Ae^{-k_{1}t}+(1-A)e^{-k_{2}t}\right], \end{array}$$

where $F_{p}$ is the plasma flow, $v_{e}$ is the extravascular extracellular volume fraction, $v_{p}$ is the blood plasma volume fraction, and the sum $v_{e}+v_{p}$ is the total extracellular volume fraction. Here A, $k_{1}$, and $k_{2}$ are free parameters from which estimates of $v_{e}$, $v_{p}$ and the capillary permeability surface area product, PS, can be determined. We reparameterize $\left(F_{+},F_{-}\right)$ in terms of $\left(F_{p},A\right)$ yielding a bounded parameter A∈[0,1] which is more convenient for model fitting and better aligned with the TK model parameterization.

These are nested models [16]; the 2CXM collapses to the same mathematical form as the TK model when A=1 or 0, or $k_{1}=k_{2}$.

*Blind deconvolution*: Blind deconvolution was performed according to [12], using the Perflab web application [14], which provides an implementation of model‐constrained, multi‐channel alternating minimization. Initialization was performed using the representative AIF [2]. To account for non‐constant temporal resolution, AIF and tissue models were evaluated on an equidistant temporal grid (using the shortest sampling interval in the data) and an additional nearest‐neighbor resampling step was applied when evaluating residuals against measured (non‐uniformly sampled) tissue data.

*Clustering*: Blind deconvolution relies solely on tissue data, which must be divided into representative sub‐regions or channels; this can be done automatically through clustering [5]. Clusters define a group of voxels. A channel is a single concentration curve, defined as the mean concentration curve over the voxels in a cluster.

The whole process was implemented in the Perflab software [14]. Input concentration curves from tumors were used. Each voxel time‐series was normalized to have zero mean and unit variance. From the normalized signals, several features were extracted, including maximum, skewness, kurtosis, skewness of the magnitude spectrum, kurtosis of the magnitude spectrum, and time to peak. Subsequently, k‐means clustering with up to 30 clusters was performed. The clustering was initialized with 10 restarts from a single fixed seed. To select the optimal number of clusters, the silhouette score was used [19]. After determining the optimal number of clusters, cluster labels were used to extract the original concentration curves from the dataset, which were then averaged according to the labels to form the respective channels to be later used in the blind deconvolution.

### 2.2 Data Acquisition & Preparation

*Data acquisition*: The study was approved by a local research ethics committee and written informed consent was obtained from each patient. Forty patients were scanned on a 1.5 T Siemens Aera MR scanner using a bilateral breast coil [20]. In 30 of those patients, an additional flexible matrix coil was placed on the back to improve the aortic signal and our analysis was restricted to this group of 30. $T_{1}$ maps were generated before and after the dynamic sequence using an inversion recovery (IR) prepared spoiled gradient echo sequence with four inversion times. The dynamic series consisted of 93 high temporal resolution volumes ($T_{1}$‐weighted 3D FLASH, temporal resolution ≈2 s), interleaved with eight high spatial resolution volumes (in this study used only for tumor ROI delineation). The FOV encompassed both breasts, the heart, the aortic arch, and part of the descending aorta. Full acquisition details are provided in [2].

*Post‐processing & direct AIF measurement*: The signal intensity (SI)‐time data were converted to CA concentration using bookend $T_{1}$ measurements [21] assuming fast water exchange between tissue compartments [22]. ROIs were manually drawn inside the descending aorta, following the procedure of [2], to obtain aortic AIFs. Tumor ROIs were delineated according to the procedure described in [20].

SNR was calculated for each patient using the mean SI‐time curve within the whole tumor ROI. Specifically, SNR was defined as

(5) $$\text{SNR}=\frac{\text{mean}({\text{SI}}_{\text{baseline}})}{\text{std}({\text{SI}}_{\text{baseline}})},$$

where ${\text{SI}}_{\text{baseline}}$ denotes the baseline (pre‐contrast) signal intensities.

### 2.3 AIF Scaling and Comparison

CO is inversely proportional to the area under the first‐pass peak of the AIF [6, 15]. Because CO should be identical for aortic and local AIFs from the same patient, we can exploit this relationship to scale the blind AIFs in order to isolate the shape of the AIFs for comparison. In practice this is not a feasible method of scaling in a study without a measurement of the AIF or patients CO. In this study, the area under the first‐pass peak was calculated from the aortic AIF and applied as a scaling factor to the blind AIFs, after the blind deconvolution was completed.

For this study we define scale‐invariant metrics to quantify the shape characteristics of AIFs in a way that is robust to the chosen scaling method. These metrics were selected to be independent of each other, and to capture complementary aspects of the AIF. In addition, we define scale‐dependent metrics whose purpose is to assess the reliability of the CO‐based scaling. The following scale‐invariant measures were used:

*Arterial dispersion (AD [s])*: AD was defined as the ratio of the area under the arterial first‐pass peak to the maximum value at the peak, providing a measure of the peak width and thus of AD [1, 13, 23].

*Normalized peak magnitude (NPM [a.u.])*: NPM was introduced to quantify the prominence of recirculation peaks throughout an AIF. To calculate this metric, the AIF model was evaluated without the tri‐exponential decay, the signal was trimmed to begin at the second‐pass peak, normalized to zero mean and unit variance, and NPM was defined as the mean absolute value of the resulting signal. NPM can be interpreted as a measure of how much time the normalized signal spends away from 0 and near its peak values. Signals with more prominent, sustained peaks therefore have higher NPM, whereas signals whose peaks disperse towards zero have lower NPM. By Cauchy–Schwarz NPM can be shown to be bounded between 0 and 1.

The following scale‐dependent measures were used: Tail [mM] was defined as the average value of the AIF at the final five time points, and the area under the whole AIF was denoted ${\text{AUC}}_{\text{whole}}$ [mM·s].

### 2.4 Metric Thresholds

Local AIFs are expected to be more dispersed than aortic AIFs; therefore, direct comparison of AD and NPM is not appropriate. Instead, one‐sided thresholds were defined to assess whether local AIFs exhibit physiologically plausible levels of dispersion. Increased dispersion is expected to reduce structure in the AIF, resulting in higher AD and lower NPM. However, $T_{2}$* effects may lead to underestimation of the peak signal of the measured AIF by up to 10% [24]. Propagating this through the signal‐concentration relationship corresponds to an estimated ≈14% underestimation in CA concentration at the peak. On average, AD of the corrected AIFs would be ≈8% shorter than that of the measured AIFs.

Based on this, a 10% one‐sided threshold was applied to AD and NPM; the blind AIF is allowed to have an AD that is 10% less than the measured AIF and an NPM that is 10% larger than the measured AIF.

The robustness of the results to this choice of threshold was assessed using a sensitivity analysis over threshold values ranging from 0% to 20% in increments of 1%.

## 3 Results

### 3.1 Data Quality/Channels

Of the 30 patients analyzed, two patients were excluded due to missing IR data, and a further three were excluded due to having a small tumor volume (<1 cc).

The median SNR across all patients was 22.7 with a range of 7.2–88.3. The median number of channels across all patients was 8 with a range of 5–17.

### 3.2 Blind Estimation Results

**FIGURE 1:**
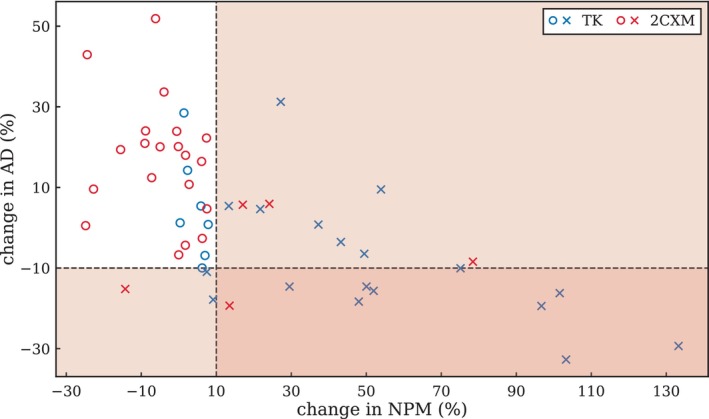
Scatter plot showing the change in AD from the measured AIF to the blind AIF plotted against the percentage change in NPM for both TK (blue) and 2CXM (red). The 10% thresholds are indicated by red regions; points exceeding these thresholds are plotted as crosses, whereas points within the acceptable range are plotted as circles.

Blind AIF estimates were obtained for all 25 patients with both the TK and 2CXM models. The estimated AD and NPM values were compared with those derived from the measured AIF. Based on the thresholds described previously, 7/25 TK and 20/25 2CXM estimates fell within the acceptable range for both AD and NPM, whereas 9/25 TK and 1/25 2CXM estimates exceeded the thresholds for both metrics. These results are illustrated in Figure 1, which shows the percentage change in AD from the measured AIF to the blind AIF plotted against the percentage change in NPM.

To assess the effect of increasing model complexity on a per‐patient basis, the threshold classifications were combined to form the contingency table shown in Table 1. Each estimate was labeled good when both AD and NPM fell within their respective thresholds, and bad otherwise. This enables a direct comparison of TK and 2CXM classifications for the same patient.

**TABLE 1:** Contingency table showing the number of cases in which TK and 2CXM produced good or bad AIF estimates. An estimate is labeled good when both AD and NPM fall within their respective thresholds.

		2CXM	
		Good	Bad
TK	Good	7	0
	Bad	13	5

A sensitivity analysis was performed across integer thresholds from 0% to 20%. A binomial logistic regression model was fitted to the number of good and bad outcomes as a function of model type and threshold, including an interaction term. This showed that both model and threshold were significant predictors of a good outcome (p<0.001), while the interaction between model and threshold was not significant (p=0.992). This indicates that although the choice of threshold affects overall performance, the relative difference between models is consistent across thresholds, with 2CXM consistently outperforming TK. Exact McNemar tests performed at each threshold confirmed that the 2CXM significantly outperformed the TK model in all cases (p<0.01 for all thresholds). Together, these results indicate that the model comparison is robust to threshold choice. Results are presented using the prespecified 10% threshold (Section 2.4).

**FIGURE 2:**
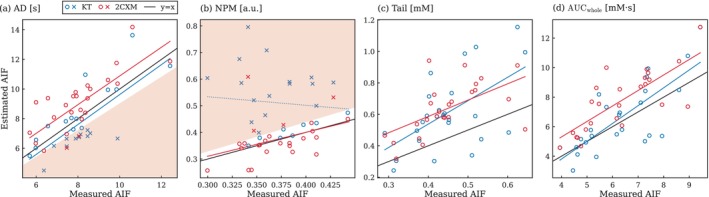
Scatter plots comparing measured and blind‐estimated AIF metrics for the TK and 2CXM models. Each point represents a patient‐specific estimate. Solid colored regression lines indicate significant correlations (p≤0.05; see Table 2); dashed colored lines indicate non‐significant correlations (p>0.05; see Table 2). The black line indicates y=x. For AD (a) and NPM (b) the 10% thresholds are indicated by red regions; points exceeding these thresholds are plotted as crosses, whereas points within the acceptable range are plotted as circles.

**FIGURE 3:**
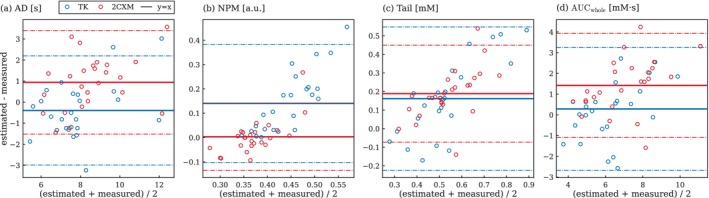
Bland–Altman plots comparing measured and blindly estimated AIF metrics for the TK and 2CXM models. Each point represents a patient‐specific estimate. Solid colored lines indicate the mean difference, and dash–dot colored lines indicate the ±1.96 SD limits of agreement. In panel (b), the mean difference of TK and upper limit of agreement for 2CXM overlap.

Measured and blind AIF metrics were then compared using scatter plots (Figure 2) and Bland–Altman plots (Figure 3). Linear regression lines indicate the presence or absence of significant correlations. The 10% thresholds for AD and NPM are denoted by the red shaded regions. Crosses are used to distinguish points that exceed the thresholds. The full regression results are presented in Table 2. Across all metrics, the blind AIF obtained using the 2CXM correlated better with the measured AIF than that obtained using the TK model. The results for each metric are described briefly below.

**TABLE 2:** Grouped regression results with slope (coefficient), Pearson correlation coefficient (r), and p‐values. For each metric, the higher r is highlighted in bold. p values ≥0.05 are shown in italic.

Metric	Model	Slope	p	r
AD	TK	1.01	<0.001	0.755
	2CXM	0.97	<0.001	**0.758**
NPM	TK	−*0.32*	*0.6570*	−*0.094*
	2CXM	0.90	0.0495	**0.397**
Tail	TK	1.49	0.0029	0.571
	2CXM	1.04	0.0028	**0.573**
${\text{AUC}}_{\text{whole}}$	TK	1.21	<0.001	0.768
	2CXM	1.05	<0.001	**0.771**

*AD*: Both TK and 2CXM AD were strongly correlated with the aortic values. However, 2CXM produced longer AD on average. *NPM*: NPM from 2CXM correlated with the measured AIF, whereas TK blind AIFs showed no correlation. *Tail*: Tail concentration was weakly correlated for both TK and 2CXM, with blind AIFs generally overestimating it compared to the measured AIF. ${\mathrm{AUC}}_{\mathrm{whole}}$: Both TK and 2CXM ${\text{AUC}}_{\text{whole}}$ were correlated with the aortic value, although 2CXM systematically overestimated it. Overall, 2CXM blind AIFs showed stronger agreement with measured AIFs across all metrics.

## 4 Discussion

We obtained estimates of the AIF for all 25 patients, using both TK and 2CXM. Across all metrics, 2CXM had a higher correlation with the measured AIF. To our knowledge this is the first study to include data where active steps were taken at the acquisition stage to measure the AIF for the purpose of validating blind estimation in clinical DCE‐MRI. While previous attempts have been made [4, 5, 9, 11], those studies either relied on AIFs acquired using sequences not specifically designed for accurate AIF measurement, or omitted measured AIFs from the analysis.

### 4.1 Modeling

We chose to compare blind estimates of the AIF derived from two different tissue models, a one‐compartment (TK) and a two‐compartment (2CXM) model. We did not employ the extended TK model despite its widespread application in the literature [25]. The extended TK model represents an intermediate approximation between the two models used [18] and would therefore be captured by the 2CXM if it were the preferred tissue model. However, when the blood volume term of the extended TK model is non‐negligible, and therefore the tissue is assumed to be highly perfused, the model is difficult to justify for the analysis of solid tumors using high temporal resolution DCE‐MRI data from either a theoretical [16, 18, 26] or practical [27, 28] perspective.

### 4.2 Data Quality/Channels

**FIGURE 4:**
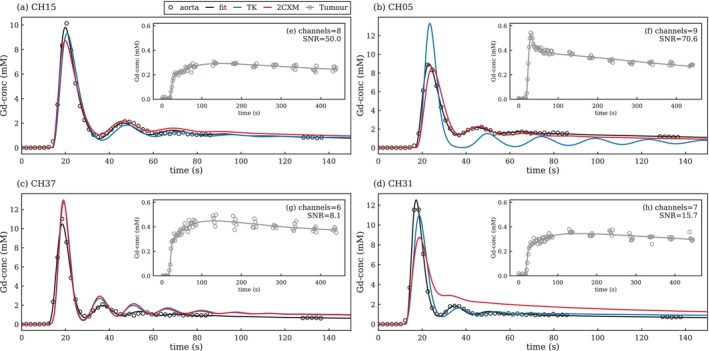
Examples of blind and measured AIFs. Panels (a–d) illustrate cases where: (a) both models meet AD/NPM thresholds; (b) only 2CXM meets them; (c) at least one metric falls outside threshold for both models; and (d) both models meet the criteria but 2CXM shows excessive dispersion. The inset axes (e–h) show the corresponding 2CXM tumor fits using the measured AIF.

The quality of the blind AIF estimates was influenced by both SNR and channel count. Even in high‐SNR cases, systematic errors may persist due to the ill‐conditioned nature of the blind deconvolution problem [12]. Across the patients, the median SNR was 22.7, with channel counts ranging from 5 to 17 (see Section 3.1). For example, the blind AIFs produced by TK and 2CXM in patient CH37 (SNR 8.1, six channels) both had metrics which did not meet the thresholds (Figure 4c). The corresponding tissue curve in Figure 4g illustrates the combination of low SNR and low channel count, which likely caused this poor performance. Patients with low SNR and fewer channels, and potentially with limited channel diversity [7], tended to exhibit poorer blind AIF estimates. Channel diversity was not explicitly quantified in this study but was qualitatively assessed through visual inspection of the individual channel signals. A more quantitative assessment of channel diversity may help clarify its role in future studies.

### 4.3 Metric Interpretation

The contingency‐table grouping (Table 1) highlights a clear trend: 2CXM generally outperforms TK in blind deconvolution. This behavior is consistent with the expected dispersion of downstream AIFs, where increased AD and decreased NPM reflect physiological dispersion. In line with this expectation, 2CXM typically produced longer AD and NPM values that were similar to, and strongly correlated with, those derived from the measured AIF.

To illustrate these behaviors, Figure 4 presents four representative examples corresponding to the regimes summarized in Table 1. In each case, the inset shows the corresponding 2CXM fit to the mean concentration curve across the whole tumor using the measured AIF.

When both models recover physiologically plausible AIFs (Figure 4a), the resulting curves are visually similar to each other and to the measured AIF. However, in cases where the tissue curve exhibits a pronounced vascular phase (Figure 4b), TK fails to meet the AD and NPM thresholds. The resulting TK AIF displays a tall, narrow first‐pass peak and pronounced recirculation peaks. We interpret this as a consequence of the blind deconvolution process compensating for the limited flexibility of the TK model. The blind deconvolution therefore introduces this missing structure into the estimated AIF, leading to AIFs with artificially sharp peaks and prominent recirculation peaks in the tail. These effects are reflected quantitatively by a decrease in AD and an increase in NPM. In more challenging conditions, such as low SNR or reduced channel count (Figure 4c), both models can deviate substantially from the measured AIF despite producing similar blind estimates.

Finally, Figure 4d highlights a limitation of the grouping scheme. Although, both TK and 2CXM meet the AD and NPM criteria, the 2CXM blind AIF is very dispersed relative to the measured AIF, whereas the TK blind AIF is not. This occurs because the grouping only constrains AD and NPM in one direction and does not penalize excessive dispersion of the AIF. We hypothesize that this could arise from the combination of low SNR and a low channel count, which may provide insufficient information for a more complex model such as 2CXM to converge to a stable solution. In this example, the mean tissue curve is also relatively simple, with no visible first‐pass peak, and the TK model can therefore describe the data well without introducing spurious structure. However, we cannot rule out that the 2CXM AIF is closer to the true underlying AIF which is the reason we place one‐sided thresholds.

Together, these observations indicate that while 2CXM is generally the more reliable option for blind AIF estimation, its advantages depend strongly on the underlying data. When the tissue curves lack a pronounced vascular phase (which avoids a key limitation of the TK model), TK has the potential to outperform 2CXM in cases with low SNR or in homogeneous tumors.

### 4.4 Cardiac Output Scaling

The overestimates in both scale‐dependent metrics, tail and ${\text{AUC}}_{\text{whole}}$, suggest that alternative scaling methods, such as scaling by a measured AIF tail, may need to be sought [4]. This is further supported by the fact that CO scaling is very sensitive to changes in the first‐pass peak, such as underestimation of the peak signal due to $T_{2}$* effects, or overestimation as a result of blind estimation (as we see in the TK AIF for Figure 4b).

### 4.5 Clinical Implications and Limitations

Local AIFs should better reflect the input to the tissue of interest, compared to a global AIF sampled upstream (e.g., from the aorta). Our findings suggest that blind estimation can provide such local AIFs, although the accuracy remains dependent on the number of channels, SNR, and tissue model choice.

Several limitations of this study should be acknowledged. First, blind estimation depends strongly on the clustering step, and different choices of clustering algorithm or number of channels could affect the outcome. Second, there is no gold standard for the measured AIFs. They may suffer from $T_{2}$* effects causing the peak signal to be underestimated; however, steps were taken to minimize potential errors [2]. Finally, while the Horsfield model is flexible enough to produce AIFs with many visible recirculation peaks, this flexibility may encourage overfitting; a problem simpler AIF models may be less susceptible to.

These limitations highlight the need for further validation studies and careful consideration of clustering techniques.

### 4.6 Conclusions

In this work, we evaluated blind estimation of the AIF using TK and 2CXM models and showed that 2CXM generally provides more reliable estimates of AIF structure and dispersion. Our findings also demonstrate that model performance depends on data quality and tissue characteristics, indicating that model selection should be informed by these factors. However, further work is needed to determine how these factors can be used systematically to guide model selection in practice.

In addition, our results suggest that cardiac output scaling may overestimate the amplitude of the blind AIF. If such limitations can be addressed, blind local AIF estimation could support more accurate perfusion modeling in clinical DCE‐MRI, where direct measurement of the AIF is not feasible.

## Funding

This work was supported by Breast Cancer Now (award 2014MayPR241), the Engineering and Physical Sciences Research Council (Grant No. EP/S022732/1), the Ministry of Education, Youth and Sports of the Czech Republic (Large RI Project LM2023050 Czech‐BioImaging), and the Czech Science Foundation (Grant No. GA22‐10953S).

## Figures and Tables

**FIGURE A1:**
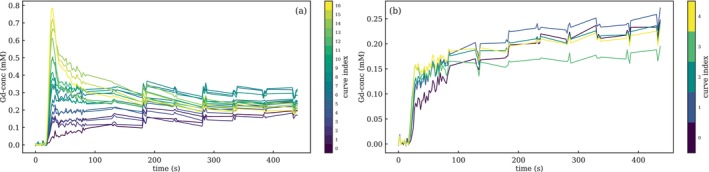
Tissue concentration‐time curves for Patient 18 (a) and Patient 36 (b), with 17 and five channels, respectively.

**FIGURE A2:**
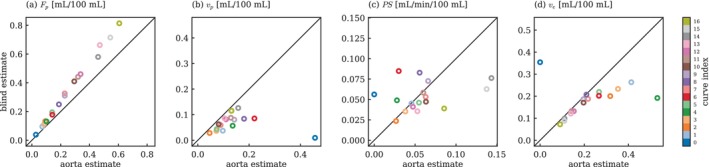
2CXM parameter estimates for the 17 channels of Patient 18. The fits to curve zero are effectively both TK models, leaving ambiguity about the assignment of $v_{e}$ and $v_{p}$ ($v_{e}=0$ using the measured AIF and $v_{p}=0$ using the blind AIF).

**FIGURE A3:**
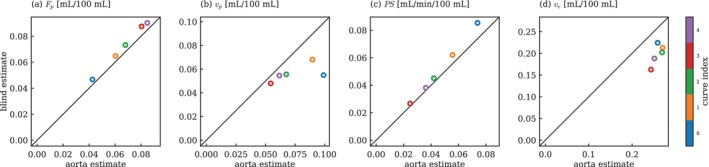
2CXM parameter estimates for the five channels of Patient 36.

## Data Availability

The data that support the findings of this study are available from the corresponding author upon reasonable request.

## Appendix A

To illustrate the diversity of tissue curves, we present two representative examples corresponding to patients with a high and low number of tissue channels (Figures A1, A2, A3).

For each example, we compare 2CXM parameter estimates ($F_{p}$, $v_{p}$, PS, and $v_{e}$) obtained using the measured AIF against those derived using the blind AIF. Scatter plots show the measured AIF estimates on the *x*‐axis and blind AIF estimates on the *y*‐axis. Results based on TK blind AIFs are omitted.
